# Ppb-level detection of methane based on an optimized T-type photoacoustic cell and a NIR diode laser

**DOI:** 10.1016/j.pacs.2020.100216

**Published:** 2020-12-16

**Authors:** Zhenfeng Gong, Tianli Gao, Liang Mei, Ke Chen, Yewei Chen, Bo Zhang, Wei Peng, Qingxu Yu

**Affiliations:** a School of Optoelectronic Engineering and Instrumentation Science, Dalian University of Technology, Dalian, 116024, Liaoning, China; b Tbea Electric Co., Ltd., Changji, 831100, Xinjiang, China; c School of Physics, Dalian University of Technology, Dalian, 116024, Liaoning, China

**Keywords:** T-type resonant photoacoustic cell, Fiber-optic acoustic sensor, Photoacoustic spectroscopy, Methane

## Abstract

This paper presents an optimized T-type resonant photoacoustic (PA) cell for methane (CH_4_) gas detection. The noise transmission coefficients and PA field distributions of the T-type resonant PA cell have been evaluated using the finite element method and thermoviscous acoustic theory. The optimized T-type resonant PA cell, together with a near-infrared (NIR) distributed feedback (DFB) laser source, a high-speed spectrometer and a fiber-optic acoustic sensor constitutes a PAS system for CH_4_ detection. The sensitivity is measured to be 1.8 pm/ppm and a minimum detectable limit (MDL) of 9 parts per billion (ppb) can be achieved with an averaging time of 500 s. The optimized T-type longitudinal resonant PA cell features of high PA cell constant, fast response time and simple manufacturing process.

## 1 Introduction

Methane (CH_4_) plays an important role in the monitoring of environmental pollutant [1,2], safety in coal mines [3,4], medical diagnostics [5,6] and dissolved gases analysis in transformer oil [7,8]. In the atmosphere, there is a certain concentration of CH_4_, which causes the greenhouse effect as well as carbon dioxide (CO_2_). Therefore, it is very important to realize the accurate and high-sensitivity detection of CH_4_. Conventional methods such as electro-chemistry [9], semiconductor [10], and catalyst combustion [11] are widely employed for CH_4_ detection. However, these methods have one or more disadvantages such as low sensitivity and selectivity, frequent calibrations requirement, system complexity and high cost. Recently, optical methods based on absorption spectroscopy such as infrared tunable diode laser absorption spectroscopy (TDLAS) [7,12], cavity ring down spectroscopy (CRDS) [13,14], and cavity enhanced absorption spectroscopy (CEAS) [15,16], have been used for trace methane detection. Dong et al. used an interband cascade laser (ICL) as the excitation source and a methane detection limit of 5 ppb (parts per billion) was achieved based on TDLAS [12]. However, the ICL and multipass gas cell employed in this system were sophisticated and of high cost, which limited the applications, mainly in scientific fields.

Photoacoustic (PA) spectroscopy (PAS) has been one of the most versatile methods for trace gas analysis because of high sensitivity and selectivity [6,8,[17], [18], [19], [20], [21], [22], [23], [24], [25]]. In recent years, a lot of efforts have been made to optimize the PA systems by the researchers. Quartz enhanced photoacoustic spectroscopy (QEPAS) employs a piezoelectric quartz tuning fork (QTF) to act as an acoustic detector [[26], [27], [28], [29], [30]]. Hu et al. proposed a QEPAS based methane sensor based on a QTF-embedded, double-pass and off-beam configuration [30]. A 1σ detection limit of 8.62 ppm (parts per million) was achieved at the averaging time of 0.3 s with the second harmonic detection technique. For further improvement of the amplitudes of PA signals, cavity-enhanced photoacoustic spectroscopy (CEPAS), in which the excitation laser power is fully utilized, has been presented [[31], [32], [33]]. Wang et al. demonstrated an ultra-sensitive PA sensor based on CEPAS. By placing a PA cell inside the cavity, the laser power was enhanced by more than 630 times and an ultra-low detection limit for trace gas was achieved [33].

Besides, the optimizing of the PA cells has attracted a lot of interest by the researchers [[34], [35], [36], [37], [38], [39], [40]]. Zheng et al. designed a PA module for CH_4_ detection with a compact differential PA cell, which consisted of two acoustic resonators [40]. The PAS system has achieved a detection limit of 3.6 ppm with the averaging time of 1 s. Nevertheless, the differential PA cell was based on the conventional H-type PA cell with two buffers at both ends of the resonator. So it had a large volume, leading to the increasing of the diffusion time of CH_4_. Furthermore, an opening must be fabricated in the center of the resonator to be cooperated with the acoustic sensor, which increased the difficulty of fabricating the PA cell. In our previous work, a T-type longitudinal resonant PA cell has been developed for trace gas detection [41]. The T-type longitudinal resonant PA cell possesses a fast response time, a high PA cell constant, and a simple manufacturing process. However, the construction of the T-type resonant PA cell is not optimal, and there are few theoretical analysis about the T-type PA cell.

In this paper, the noise transmission coefficients and PA field distributions of the T-type resonant PA cell have been theoretically evaluated to optimize the construction of T-type resonant PA cell, using the finite element method and thermoviscous acoustics theory. A CH_4_ detection system, employing the optimized T-type resonant PA cell, a cheap near-infrared (NIR) distributed feedback (DFB) laser source, and a fiber-optic acoustic sensor, has been developed for CH_4_ monitoring with sub-ppb level detection sensitivity.

## 2 Optimization of the T-type PA cell

**Fig. 1:**
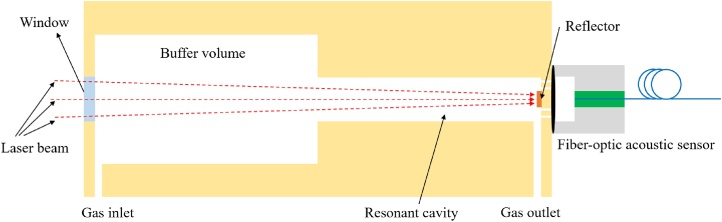
Schematic structure of the optimized T-type longitudinal resonant PA cell.

The schematics of the optimized T-type longitudinal resonant PA cell is shown in Fig. 1. The proposed optimized T-type resonant PA cell consists of a resonant cavity, a buffer volume, a window, a reflector, a gas inlet and a gas outlet. The fiber-optic acoustic sensor is placed at the end of the resonator, where is the anti-node position of the PAS signals. In our previous work [41], the length of the buffer volume was half of the resonant cavity as the traditional H-type resonant PA cell, which was not been optimized by theoretical analysis. However a suitable construction of buffer volume can deduce the noise coming from the window in the PA cell and prevent the ambient noise outside the PA cell from coupling into the PA cell. Furthermore, the Q factor the PA cell is related with the buffer volume. So it is very important to precisely optimize the construction of the buffer volume.

Assuming that the cross-sectional areas of the resonant cavity and the buffer volume are *S_1_* and *S_2_*, respectively, and the length of the buffer volume is *L*. The window is located at *x = 0*, and the cross-sectional area of the window is *S_1_*. The boundary between the resonant cavity and the buffer volume is located at *x = L*. The acoustic pressure *(p_i_)_1_* and velocity *(v_i_)_1_* of the ambient noise can be given as:

(1) $${\left(p_{i}\right)}_{1}=A_{1}e^{j(\omega t-kx)}$$

(2) $${\left(v_{i}\right)}_{1}=C_{1}e^{j(\omega t-kx)}$$

where *k=ω/c*, *c* is the speed of acoustic pressure. Because the trace gas in the PA cell has a very low concentration, the medium outside and inside the PA cell are considered the same approximately. *A_1_* and *C_1_* are complex constant representing the complex pressure amplitude and velocity amplitude, respectively. When the incident wave of the ambient noise first arrives at the window, where *x = 0*, some of the energy is reflected and some is transmitted into the buffer volume. After passing through the buffer volume a part of this transmitted wave is reflected at *x = L* and is returned to *x = 0*, where it is again partially reflected, the reflected portion combining with the wave being initially transmitted into the buffer volume, and the transmitted portion combining with the wave being initially reflected at *x = 0*. This process is then repeated, and after a sufficient number of transits in the buffer volume the conditions will reach a steady state, in which the rate at which energy is reflected back outside the window plus the rate at which it is transmitted into the resonant cavity are equal to the rate of arrival of the incident energy. Under these steady-state conditions the acoustic pressure and velocity of the wave reflected outside the window may be represented by:

(3) $${\left(p_{r}\right)}_{1}=B_{1}e^{j(\omega t+kx)}$$

(4) $${\left(v_{r}\right)}_{1}=D_{1}e^{j(\omega t+kx)}$$

where *B_1_* and *D_1_* are complex constant representing the complex pressure amplitude and velocity amplitude of the wave reflected outside the window, respectively. The acoustic pressure of the transmitted and reflected waves in buffer volume can be given by:

(5) $${\left(p_{t}\right)}_{2}=A_{2}e^{j(\omega t-kx)}$$

(6) $${\left(p_{r}\right)}_{2}=B_{2}e^{j(\omega t+kx)}$$

And the acoustic velocity of the transmitted and reflected waves in buffer volume can be expressed as:

(7) $${\left(v_{t}\right)}_{2}=C_{2}e^{j(\omega t-kx)}$$

(8) $${\left(v_{r}\right)}_{2}=D_{2}e^{j(\omega t+kx)}$$

The acoustic wave transmitted into the resonant cavity starts at *x*=*L*, so the coordinate origin should be shifted to the left by *L*. Therefore, the acoustic pressure and velocity of the wave transmitted into the resonant cavity is represented as:

(9) $${\left(p_{t}\right)}_{3}=A_{3}e^{j\left[\omega t-k(x-L)\right]}$$

(10) $${\left(v_{t}\right)}_{3}=C_{3}e^{j\left[\omega t-k(x-L)\right]}$$

The acoustic pressure is continuous at *x = 0* and *x = L*:

(11) $$A_{1}+B_{1}=A_{2}+B_{2}$$

(12) $$A_{2}e^{-jkL}+B_{2}e^{jkL}=A_{3}$$

And the particle velocities at *x = 0* and *x = L* are also continuous:

(13) $$S_{1}(C_{1}+D_{1})=S_{2}(C_{2}+D_{2})$$

(14) $$S_{2}(C_{2}e^{-jkL}+D_{2}e^{jkL})=S_{1}C_{3}$$

The ambient noise is a kind of plane wave, therefore *C* and *D* can be given as:

(15) $$C_{1}=\frac{A_{1}}{\rho c}$$

(16) $$D_{1}=-\frac{B_{1}}{\rho c}$$

(17) $$C_{2}=\frac{A_{2}}{\rho c}$$

(18) $$D_{2}=-\frac{B_{2}}{\rho c}$$

(19) $$C_{3}=\frac{A_{3}}{\rho c}$$

where *ρ* is the density of the air and CH_4_/N_2_ mixture. Because the concentration of the detecting CH_4_ is at ppm level, the densities of the air and the CH_4_/N_2_ mixture in our experiments are considered approximately to be the same. Bringing the Eqs. (15), (16), (17), (18), (19) into Eqs. (11), (12), (13), (14), the sound transmission coefficient becomes:

(20) $$t_{p}=\left|\frac{A_{3}}{A_{1}}\right|=\frac{2}{{\left[4\cos^{2}kL+{\left(S_{12}+S_{21}\right)}^{2}\sin^{2}kL\right]}^{1/2}}$$

where *S_12_=S_2_/S_1_*, *S_21_=S_1_/S_2_*. So the noise transmission coefficient can be expressed as:

(21) $$t_{I}=\frac{I_{3}}{I_{1}}=\frac{{\left(A_{3}\right)}^{2}/2\rho c}{{\left(A_{1}\right)}^{2}/2\rho c}=\frac{4}{4\cos^{2}kL+{\left(S_{12}+S_{21}\right)}^{2}\sin^{2}kL}$$

**Fig. 2:**
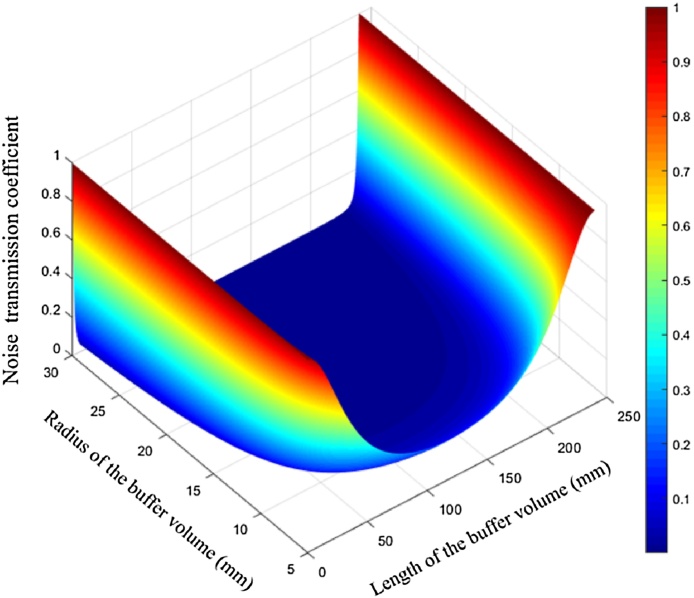
The noise transmission coefficients at different lengths and radii of the buffers according to formula analysis.

Fig. 2 shows the noise transmission coefficients at different lengths and radii of the buffers according to Eq. (21). For a fixed radius of the buffer volume, the minimum noise transmission coefficient comes up when the length of the buffer volume is 120 mm, which is equal to the length of the resonator.

**Fig. 3:**
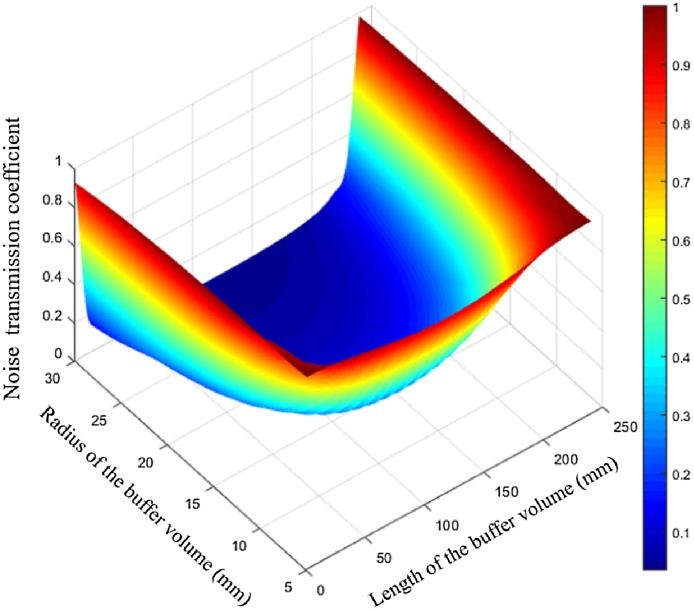
The noise transmission coefficients at different lengths and radii of the buffers according to numerical calculations.

In order to support the theoretical results of formula analysis, a 3D finite element model based on the predefined acoustic module of COMSOL Multiphysics was built to analyze the noise transmission coefficients at different lengths and radii of the buffers. A plane wave with the amplitude of 1 Pa was applied to the window on the buffer to simulate the noise signals resulting from the window. The acoustic pressure spreading into the resonator, with different lengths and radii of the buffer volumes, was recorded. As shown in Fig. 3, the simulated results have the same tendency with Fig. 2. The minimum noise transmission coefficient occurs when the length of the buffer is 120 mm.

**Fig. 4:**
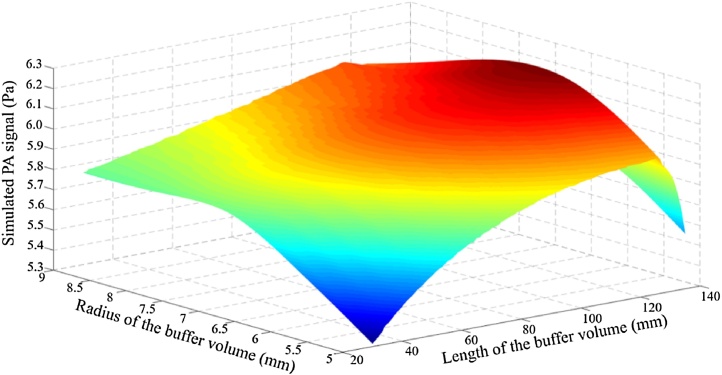
Relationships between the biggest PA signals in resonator and the geometrical parameters of the buffer volume.

**Fig. 5:**
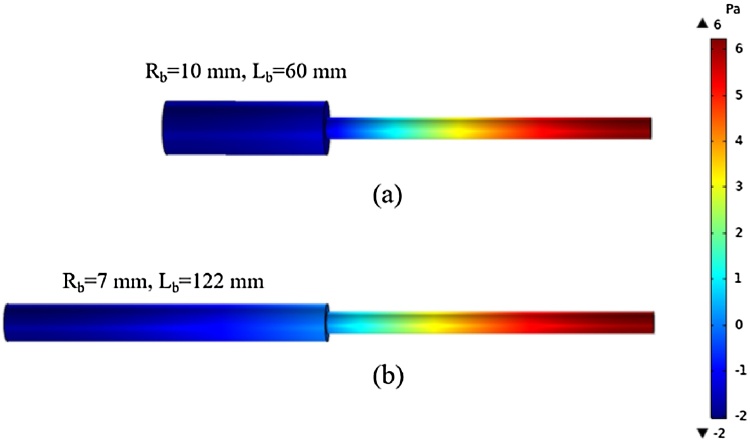
Simulated PA field distribution cloud maps of original (a) and optimized (b) T-type resonant PA cells.

In order to optimize the radius of the buffer volume, the PA field distributions at the first resonant frequency of the T-type resonant PA cell were evaluated by constructing a 3D finite element model based on COMSOL Multiphysics. In order to build a model that is matched with the experimental system, the heat loss and viscosity loss must be considered, which belonged to the category of thermoviscous acoustics. The simulated PA signals at the position of anti-node were recorded, with different inner diameters and lengths of the buffer volume. The inner radius and length of the resonator were fixed at 4 mm and 120 mm, respectively, which have been proved to be highly efficient in our previous work [41]. The relationships between the PA signals at the position of anti-node and the geometrical parameters of the buffer volume are shown in Fig. 4. As shown in Fig. 4, as the length of the buffer volume changes from 30 mm to 130 mm, the PA signal increases initially then reduces. While for the radius of the buffer volume varying from 5 mm to 9 mm, the PA signal gets bigger and then it gets smaller. The biggest simulated PA signal is presented when the radius and length of the buffer volume are 7 mm and 122 mm, respectively. That means the PA system has a best performance when the length of the buffer volume is approximately equal to the length of the resonant cavity, which is matched with the numerical results considering the noise transmission coefficient. And the radius of the buffer volume is about the twice as the resonant cavity. This can be understood as follows: if the radius of the buffer volume *R_b_* is much larger than the radius of the resonant cavity *R_r_*, most of the acoustic standing wave is limited in the resonant cavity. While a too small *R_b_* results in the decreasing of the ratio of acoustic volume over wall surface, which may lead to a lower value for the Q factor. Therefore, at suitable radius (*R_b_≈2R_r_*) a better coupling between resonator and buffer volume distributes the energy of the standing wave over both. The original finite element model and the optimized finite element model are shown in Fig. 5. The radius and length of the original buffer volume are 10 mm and 60 mm, respectively. While the optimized T-type PA cell have the radius of 7 mm and the length of 122 mm. Compared with the first-proposed T-type resonant PA cell, the optimized T-type resonant PA cell has a longer and thinner buffer volume.

**Fig. 6:**
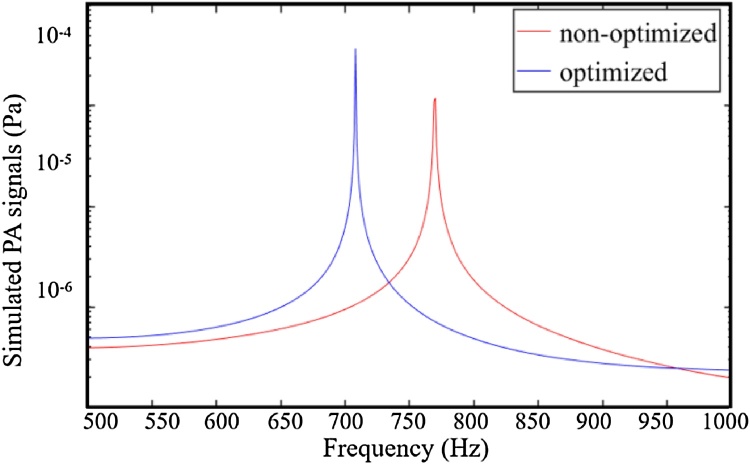
Simulated frequency responses of the optimized and non-optimized T-type resonant PA cell.

Fig. 6 shows the simulated frequency responses of the optimized and non-optimized T-type resonant PA cell using the finite element method. The simulated first-order resonant frequency of the optimized PA cell is about 707 Hz, which is completely consistent with the theoretical result based on formula analysis in our previous work [41]. Compared with the non-optimized T-Type resonant PA cell, the optimized PA cell has a better response at the first-order resonant frequency.

## 3 Experimental system and results

**Fig. 7:**
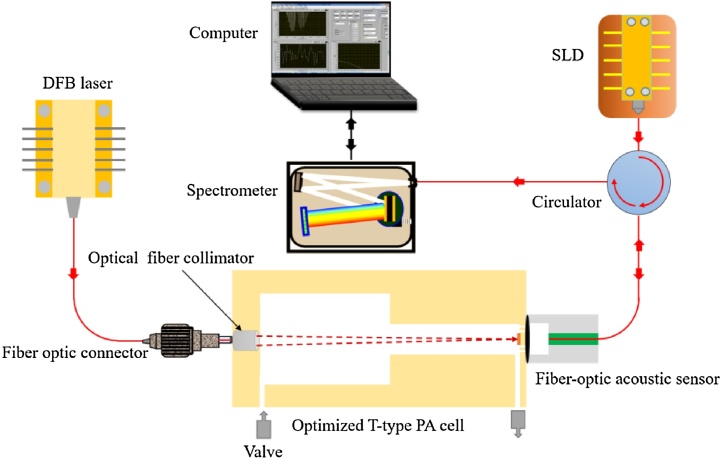
Schematic structure of the experimental setup for CH_4_ gas based on PAS.

The schematic diagram of the experimental system based on PAS is shown in Fig. 7. It consists of a NIR DFB laser source, a high-speed spectrometer, an optical fiber collimator, an optimized T-type PA cell, a fiber-optic acoustic sensor, a superluminescent light diode (SLD), a circulator and a computer. In view of the cross interference with CO_2_ and H_2_O, the characteristic spectrum line of 1650.96 nm was selected [42]. A 20 mW NIR DFB laser emitting at around 1651 nm was used as the PA excitation source. The laser beam passed into the optimized T-type PA cell through an optical fiber collimator (F260FC, Thorlabs). A high-sensitive cantilever-based fiber-optic acoustic sensor with the first-order resonant frequency of 1150 Hz was employed to detect the PA signals. The detailed description of the cantilever microphone can be found in reference [41]. There are two valves at the gas inlet and gas outlet. When the system operates, the two valves were turned off to assure airtightness of the PA cell. A SLD (DL-CS5077, Denselight) with a central wavelength of 1550 nm was used as the probe light source. The laser beam was first coupled into a circulator, and then launched into the fiber-optic acoustic sensor. The generated PA signal deformed the cantilever and leaded to the change of the Fabry-Perot (FP) cavity length. The lock-in white-light interferometry (WLI) demodulation algorithm was used to demodulate the dynamic cavity length of the fiber-optic acoustic sensor.

**Fig. 8:**
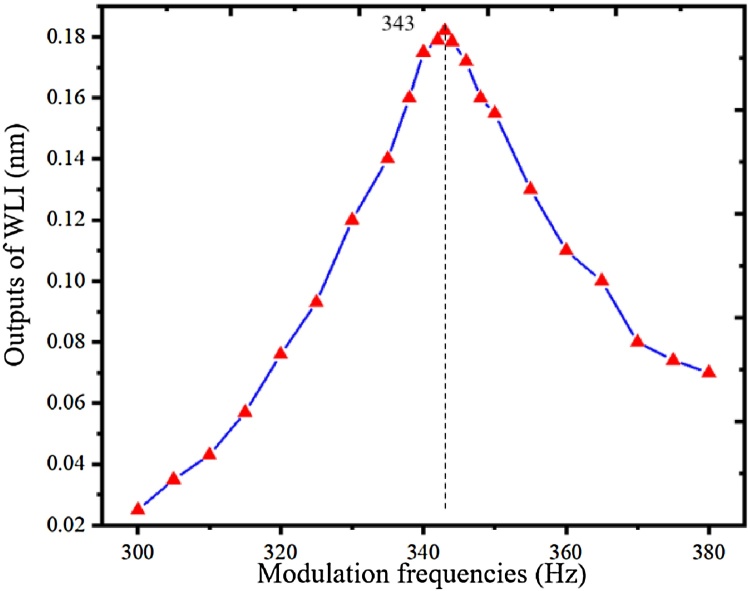
Experimental frequency responses of the optimized T-type resonant PA cell.

The first-order resonant frequency of the optimized T-type resonant PA cell was measured experimentally. The CH_4_ with the concentration of 100 ppm was flowed into the PA cell, and the modulation frequencies were changed from 300 Hz to 380 Hz. The frequency responses were obtained using the second-harmonic wavelength modulation spectroscopy (2f-WMS) technique. According to Fig. 8, there is a peak value at the modulation frequency of 343 Hz, therefore the first-order resonant frequency of the optimized T-type resonant PA cell is about 686 Hz, which is close to the simulated and theoretical analysis results. Compared with the theoretical frequency response shown in Fig. 6, the Q-factor is lower. The main reason is that in the theoretical results, the damping and loss effects were ignored in order to simplify analytical model.

**Fig. 9:**
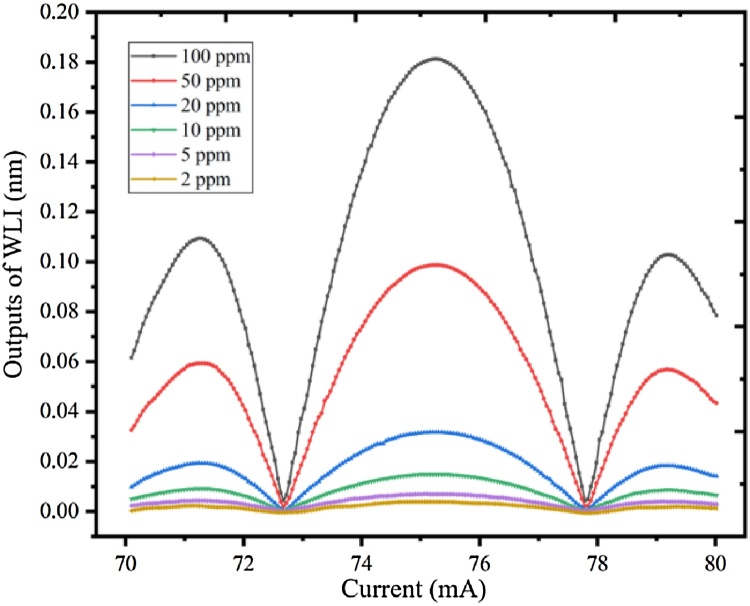
2f-WMS signals with different concentrations of CH_4_/N_2_ gas mixture.

**Fig. 10:**
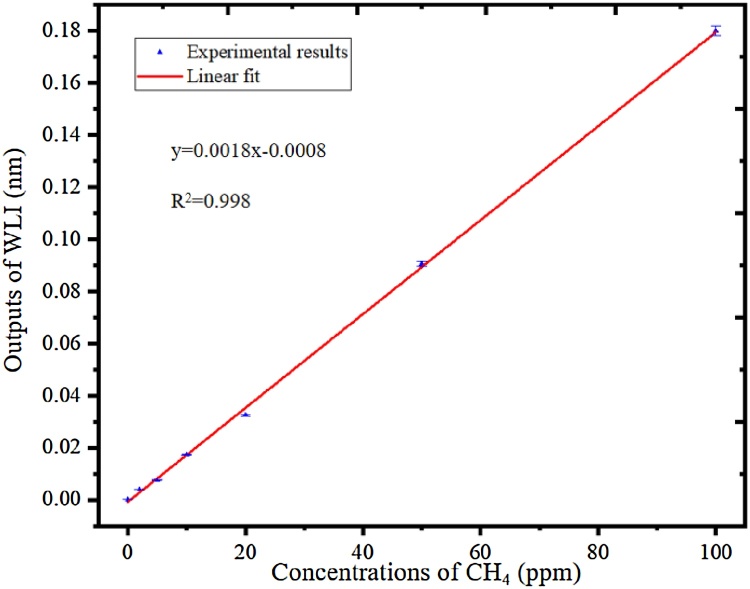
The PAS signals as a function of CH_4_ concentrations.

In order to further verify the feasibility of the CH_4_ detection system, the CH_4_ gases with different concentrations have been experimented. Two gas flowmeters (D07-19, SevenStar) with an error of ±1 %, made up of a gas mixing system to generate different concentrations of CH_4_/N_2_ gas mixture. Two bottles of CH_4_/N_2_ mixture with 10.2 ppm and 101 ppm, which were approximately equal to 10 ppm and 100 ppm, are used as the standard gases. Various CH_4_/N_2_ mixture from 2 ppm to 100 ppm were flowed into the PA cell. The laser bias was increased from 70 mA to 80 mA. Fig. 9 shows the 2f-WMS signals of CH_4_ with the concentrations of 5 ppm, 10 ppm, 20 ppm, 50 ppm and 100 ppm, and Fig. 10 shows the generated PAS signals as a function of CH_4_ with different concentrations with the responsibility of 1.8 pm/ppm by linear fitting. The linear fitting R square is >0.998, which verifies the linearity of PAS system response to different concentrations of CH_4_.

**Fig. 11:**
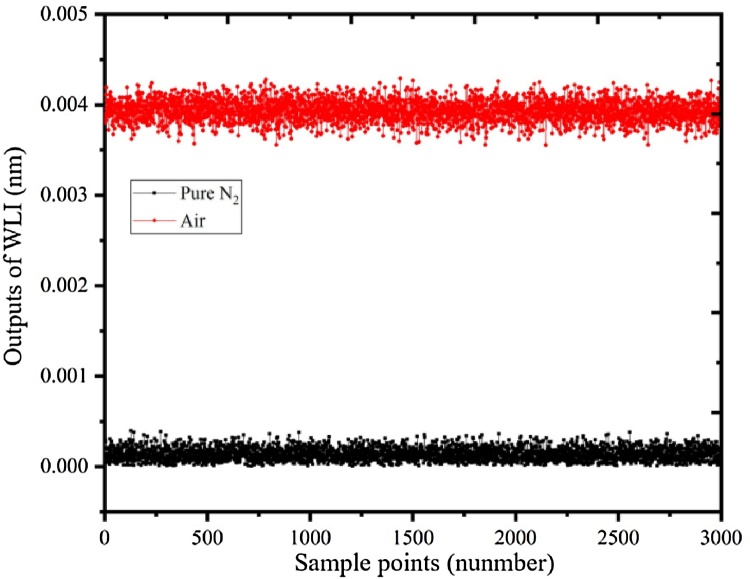
The background noise of the optimized T-type resonant PA cell by filling the PA cell with pure N_2_ and air.

**Fig. 12:**
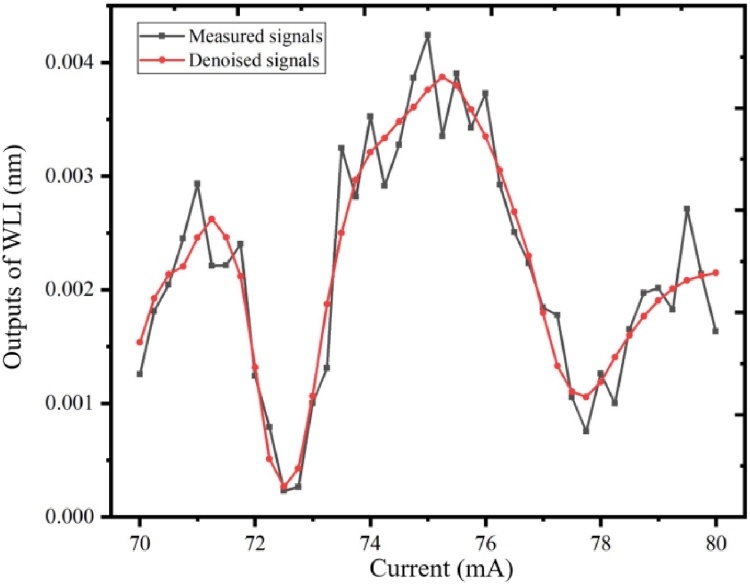
2f-WMS signals of the CH_4_ in the air.

By filling the PA cell with pure N_2_, the background noises were achieved with the integration time of 10 s, which is shown in Fig. 11. The air in our laboratory was flowed into the PA cell through an air pump. From Fig. 11, the 2f-WMS signals of air in our laboratory is apparently higher than the signals of pure N_2_. The 2f-WMS signals of CH_4_ in the air is shown in Fig. 12. The red line represents the processed spectrum by wavelet denoising. As shown in the Fig. 12, the denoised signals presents a good line shape and the peak value is 0.00387 nm. With the responsiveness of 1.8 pm/ppm, the concentration of CH_4_ in the air is calculated to be 2.2 ppm.

**Fig. 13:**
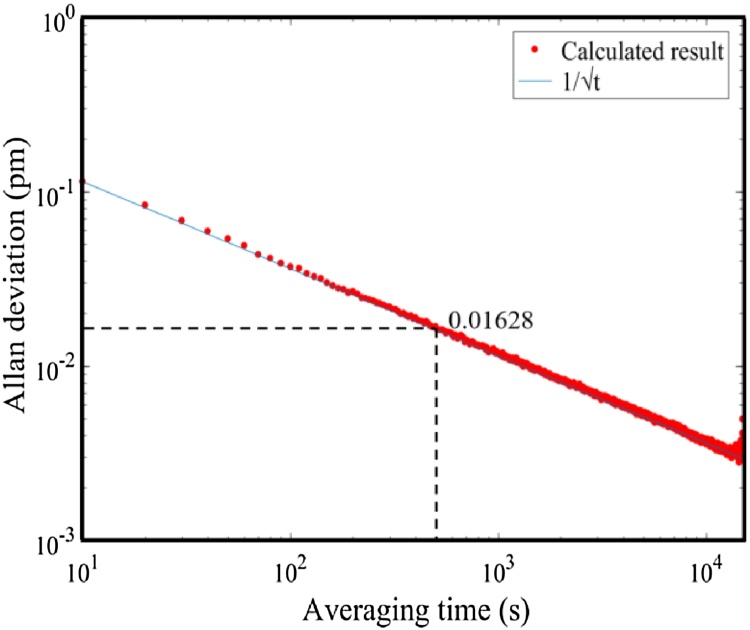
Allan deviation as a function of the data averaging period.

When the T-type resonant PA cell was filled with the air, the Allan-Werle deviation was carried out to evaluate the minimum detection limit (MDL) for trace CH_4_ detection. As shown in Fig. 13, the Allan deviation is 0.01628 pm with an averaging time of 500 s. With the responsibility of 1.8 pm/ppm, the MDL can be estimated to be 9 ppb.

## 4 Conclusion

In conclusion, we demonstrate an optimized T-type resonant PA cell. The noise transmission coefficients and PA field distributions of the T-type resonant PA cell have been evaluated to optimize the construction of the T-type resonant PA cell, using the finite element method and thermoviscous acoustic theory. A 3D finite element model based on COMSOL Multiphysics is constructed considering the heat loss and viscosity loss. The biggest simulated PA signal is presented when the radius and length of the buffer volume are 7 mm and 122 mm, respectively. The simulated first-order resonant frequency of the optimized PA cell is about 707 Hz, which is completely consistent with the theoretical result in our previous work. A 20 mW NIR DFB laser emitting at around 1651 nm is employed as the PA excitation light source. The lock-in WLI demodulation algorithm is employed to demodulate the dynamic cavity length of the fiber-optic acoustic sensor. Experimental results show that the PA signal is directly proportional to the concentrations of CH_4_. The sensitivity is measured to be 1.8 pm/ppm. The CH_4_ with the concentration of 2.2 ppm in the air is detected successfully. The MDL of CH_4_ is calculated to be 9 ppb with an averaging time of 500 s using Allan-Werle deviation.

## Funding

This work was supported by 10.13039/501100001809National Natural Science Foundation of China [grant numbers 11904045, 61905034, 61705031]; 10.13039/501100002858China Postdoctoral Science Foundation [grant numbers 2020M673542]; 10.13039/501100010018Doctoral Start-up Foundation of Liaoning Province [grant numbers 2019-BS-051]; 10.13039/501100005047Natural Science Foundation of Liaoning Province [grant numbers 2019-MS-054]; and 10.13039/501100012226Fundamental Research Funds for the Central Universities [grant numbers DUT20RC(4)014].

## CRediT authorship contribution statement

**Zhenfeng Gong:** Conceptualization, Methodology, Writing - original draft, Writing - review & editing. **Tianli Gao:** Data curation, Software, Investigation. **Liang Mei:** Software. **Ke Chen:** Funding acquisition, Methodology, Formal analysis. **Yewei Chen:** Formal analysis. **Bo Zhang:** Resources. **Wei Peng:** Funding acquisition, Project administration. **Qingxu Yu:** Visualization.

## Declaration of Competing Interest

The authors declare that there are no conflicts of interest.
